# Idiopathic Pulmonary Fibrosis: Analysis of Predisposing Variants in Patients with Familial Forms

**DOI:** 10.3390/biomedicines14010138

**Published:** 2026-01-09

**Authors:** Ilaria Stanghellini, Elena Bonora, Marco Sebastiani, Carlo Salvarani, Filippo Gozzi, Dario Andrisani, Roberto Tonelli, Nicola Rizzardi, Christian Bergamini, Federica Isidori, Marco Seri, Enrico Clini, Stefania Cerri, Olga Calabrese

**Affiliations:** 1 Medical Genetics Unit, Azienda Ospedaliero-Universitaria Policlinico di Modena, 41124 Modena, Italy; stanghellini.ilaria@aou.mo.it (I.S.); calabrese.olga@aou.mo.it (O.C.); 2 Department of Medical and Surgical Sciences (DIMEC), University of Bologna, 40138 Bologna, Italy; elena.bonora6@unibo.it; 3 IRCCS Azienda Ospedaliero-Universitaria di Bologna, 40138 Bologna, Italy; federica.isidori2@unibo.it (F.I.); marco.seri@unibo.it (M.S.); 4 Rheumatology Unit, AUSL Piacenza, 43126 Piacenza, Italy; marco.sebastiani@unipr.it; 5 Department of Medicine and Surgery, University of Parma, 43125 Parma, Italy; 6 Unit of Rheumatology, Azienda USL-IRCCS di Reggio Emilia, 42122 Reggio Emilia, Italy; carlo.salvarani@ausl.re.it; 7 Respiratory Disease Unit, Department of Medical and Surgical Sciences, University of Modena and Reggio Emilia, Azienda Ospedaliero-Universitaria Policlinico di Modena, 41124 Modena, Italy; fillo.gzz@gmail.com (F.G.); andrisanidario@gmail.com (D.A.); roberto.tonelli@me.com (R.T.); enrico.clini@unimore.it (E.C.); 8 Department of Pharmacy and Biotechnologies (FaBiT), University of Bologna, 40126 Bologna, Italy; nicola.rizzardi2@unibo.it (N.R.); christian.bergamini2@unibo.it (C.B.)

**Keywords:** idiopathic pulmonary fibrosis, CGH-SNP array, predisposition variants, NGS panel, *MUC5B* polymorphism

## Abstract

**Background**: idiopathic pulmonary fibrosis (IPF) causes progressive and irreversible changes in the lung parenchyma, leading to respiratory failure. Its pathogenesis involves several damage/repair mechanisms leading to fibrosis, whilst alterations of genes implicated in these processes contribute to the development of the disease. At present, next-generation sequencing (NGS) analyses investigate single-nucleotide or small indel variants, and no evaluation of genomic rearrangements has been so far reported. **Methods**: In order to identify predisposing variants, we analyzed—both by NGS and by comparative genomic hybridization/single-nucleotide polymorphism (CGH-SNP array) array—37 patients with a diagnosis of familial pulmonary fibrosis. **Results**: a total of 17 patients (46%) harbored copy number variations (CNVs), 10 (27%) did not harbor any CNVs, 5 (13.5%) showed a mosaic deletion of the Y chromosome, and 5 (13.5%) showed a run of homozygosity (ROH). NGS identified causative variants (including a novel one) in five patients (5/37, 13.5%) and confirmed the high prevalence of *MUC5B* promoter polymorphism rs35705950, including the detection of a previously unreported form in IPF SNP (indicated as “novel” in the main text), rs141420125 (23/37; 62%). **Conclusions**: CGH-SNP array identified CNVs containing genes involved in mechanisms (i.e., oxidative stress, mitophagy, NF-Kb pathway) that have been shown to play a role in the pathogenesis of IPF. Therefore, the application of CGH-SNP array or other quantitative tests should be considered in the diagnostic setup of these patients

## 1. Introduction

Idiopathic pulmonary fibrosis (IPF) is a progressive, chronic interstitial lung disease of unknown etiology, with a median survival of 3 to 6 years from diagnosis [1]. Although its prevalence and incidence vary according to the criteria used in the epidemiological studies carried out so far, it is still included among rare diseases, even if its incidence is rising worldwide (e.g., in Italy, the prevalence and incidence are 2.12–2.56 and 0.26–0.93 per 10,000 inhabitants, respectively [2]).

IPF results from repeated alveolar epithelial injury and aberrant fibrotic tissue repair, involving mitochondrial dysfunction, epithelial–mesenchymal transition (EMT), endoplasmic reticulum (ER) stress, cellular senescence, and excessive extracellular matrix deposition [3,4]. While several environmental and host-related factors—such as cigarette smoking, aging, gastroesophageal reflux, viral infections, and male sex—have been implicated, genetic alterations are increasingly recognized for their causal and modifying roles, particularly in familial forms of IPF (risk factors are presented by Ranzieri and colleagues [5]).

To date, two major classes of genes have been linked to IPF predisposition: pulmonary surfactant and telomerase genes. Pulmonary surfactant genes include genes encoding for surfactant proteins (SP-A, SP-B, SP-C, SP-D) and phospholipid transporters (*ABCA3*) [6]. Telomeres genes include primarily *TERT* and *TERC*, the major components of telomerase, associated with up to 15% of familial fibrosis and sporadic idiopathic cases, and secondary genes involved in the telomere assembly and maintenance processes such as *RTEL1* (telomere length regulator), *PARN* (RNase polyadenylation-specific), *NAF1*, *DKC1*, and *TINF2* [7]. In addition to surfactant proteins and telomere components, mucus is also involved in IPF predisposition, and the minor T-allele of the *MUC5B* rs35705950 promoter polymorphism (located on chromosome 11, 3 kb upstream of the *MUC5B* gene) is a strong risk factor for IPF. Initially identified in 2011 by a linkage study [8] showing that the 34% of individuals with familial forms of pulmonary fibrosis carried the rs35705950 T allele, associated with pulmonary overexpression (37.4 times higher) of *MUC5B*, subsequent studies confirmed this association in different populations and demonstrated that rs35705950 resides within an enhancer subjected to epigenetic remodeling [9].

A review of genes and common genetic variants associated with IPF risk was proposed by Yasutomo [10]. Overall, the analysis of mutations and polymorphisms in candidate genes has significantly advanced the understanding of genetic predisposition in both familial and sporadic IPF. However, alongside single-nucleotide variants, alterations such as deletions and duplications of candidate genes may also contribute to disease susceptibility, although these have thus far been reported only in animal models or isolated patients [11,12].

Given the multiple molecular pathways involved in IPF etiopathogenesis, structural variants affecting genes implicated in these processes may represent an additional, underrecognized mechanism of genetic risk.

Therefore, we adopted a comprehensive approach, combining NGS and CGH-SNP array analysis to assess, both at sequence and structural level, genetic alterations which could confer susceptibility to IPF. The aim of this study was to analyze a cohort of patients with familial IPF to search for single-nucleotide and/or CNVs s in the genes known or potentially involved (according to the processes in which they are implicated) in the etiopathogenesis of the disease.

## 2. Materials and Methods

### 2.1. Study Population

Study population was selected among the cohort of IPF patients currently followed by the Center for Rare Lung Disease of the University Hospital of Modena. IPF diagnosis was confirmed on clinical–radiological and/or pathological grounds according to current ATS/ERS/JRS/ALAT Guidelines [13]. Three patients with a diagnosis of fibrosing ILD different from IPF were also included, as they were relatives of IPF patients. Indication for genetic counseling included primarily a history of familial disease (i.e., patients with two or more affected members of the same primary biological family). Three patients without a family history of fibrosis were also included due either to early onset of the disease (2 patients: SP1356, SP1388) or to a peculiar morphological trait consisting of personal and family history of premature hair graying (1 patient: SP1198), as suggested in a recent ERS statement [14]. The counseling was performed at the Medical Genetics Outpatient Clinic of the University Hospital of Modena. Clinical and family history data were collected. Each patient provided informed consent to participate in the study. Pseudoanonymization was performed by an alphanumeric code. An aliquot of 3 ml blood sample was obtained in a tube containing EDTA. The study was approved by the Ethics Committee “Comitato Etico Area Vasta Emilia Nord (AVEN)” (Prot. AOU 000707022 del 9/3/2022), and data were treated according to the Declaration of Helsinki.

### 2.2. DNA Extraction

Genomic DNA was extracted from peripheral blood leukocytes using the Maxwell 16 System and Maxwell 16 Blood DNA Purification kit (Promega, Mannheim, Germany) according to the protocol and eluted in a final volume of 100 µL. A 1.5 μL aliquot was measured by NanoDrop One (Thermo Fisher Scientific, Waltham, MA, USA) to evaluate DNA purity and quantified by Qubit 3.0 Fluorometer (Invitrogen, Thermo Fisher Scientific, Waltham, MA, USA).

### 2.3. CGH-SNP Array

DNA samples were analyzed by CGH-SNP array, using the GenetiSure Dx Postnatal Array Kit 4 × 180 (Agilent Technologies, Santa Clara, CA, USA) CE-IVD slide, according to the manufacturer’s protocol, starting from 500 ng of DNA of the samples and reference in a volume of 20 μL. The DNA supplied in the kit was used as a control; in particular, the male control DNA was used in the case of a male patient, and the female control DNA in the case of a female one. The SureScan Dx (Agilent Technologies, Santa Clara, CA, USA) was used to scan the slides, and the TIFF images generated were analyzed by Agilent CytoDx 2.1.0.9 Software.

The GenetiSure Dx Postnatal array contains approximately 107,000 60-mer oligonucleotides copy number (CN) probes and 59,000 biallelic SNP probes. A total of 94% of the genome is covered by at least 5 CN probes per 400 kb, resulting in a median resolution of approximately 150 kb; clinically relevant regions are targeted with increased probed density, resulting in a median resolution of approximately 25 kb.

The SNP probes allow for the detection of copy-neutral changes and are distributed such that 91% of the genome have at least 100 SNP probes per 10 Mb, resulting in a median resolution for ROH of approximately 8 Mb.

CNVs and ROH were detected by Agilent CytoDx v2.2.0.4 software (Agilent Technologies, Santa Clara, CA, USA) using the default GenetiSure Dx Postnatal Analysis method_v1. Briefly, the aberration detection method algorithm 2 (ADM-2) statistical algorithm requires at least 5 contiguous suprathreshold probes (20 in the case of mosaics), a minimum average of absolute Log2 ratios (log2(Sample/Reference)) ≥ 0.25, and a minimum size (≥20 kb for gains and ≥10 kb for losses) to call CNVs. A positive log2 ratio value of +0.58 indicates trisomy (gain of 1 copy of DNA), a negative value of −1 indicates monosomy (losses of 1 copy of DNA), whereas a log2 ratio of 0 means 2 copies of DNA (diploid, normal); values higher than +0.58 may indicate mosaic gain or amplification, and values < −1 may indicate mosaic loss or complete loss (0 copies).

ROH were detected using the allele-specific copy number (ASCN) detection algorithm, which distinguishes the two alleles of an SNP by whether or not the SNP site is cleaved by the AluI/RsaI restriction enzyme mixture that is used during the sample labeling process. The algorithm constructs the distribution of log2ratio values for all SNP probes on the microarray and finds the peaks of the distribution. It fits a separate Gaussian distribution to each peak and uses a Bayesian model to calculate an expectation value for the uncut SNP allele copy number at each SNP.

The detected CNVs were classified according to the American College of Medical Genetics and Genomics (ACMG) guidelines into tiers (pathogenic, likely pathogenic, VUS, likely benign, benign) based on size, gene content, inheritance, databases, and phenotype overlap [15].

### 2.4. Real-Time Quantitative PCR (qPCR)

To confirm the CNVs identified by CGH-SNP array, primers were designed by the Primer3 tool available at the web site https://primer3.ut.ee/ (accessed 23 November 2021). Supplementary File S1 reports the primer sequences and the Tm and amplicon length of the primers designed [see Supplementary File S1]. Real-time qPCR was performed on 7500 Fast Real-Time PCR System instrument, using the PowerTrack SYBR Green Master Mix 2X (Thermo Fisher Scientific, Waltham, MA, USA) and fast-run mode PCR cycle. The amplification reactions, for the target genes and for the control, were performed in duplicate in a final volume of 15 μL containing 2X Power Track SYBR Green Master mix, forward and reverse primers at a final concentration of 500 nM, and 20 ng of DNA as template. The 2^−ΔΔCt^ method was used to assess the presence of deletions or duplications of the gene of interest. *FOXP2* was used as the control gene.

### 2.5. NGS Panel Design

For panel design, we consulted the Genomics England PanelApp, (https://panelapp.genomicsengland.co.uk/panels/, accessed 23 September 2021), and at the time of design, 24 genes were included in the Pulmonary Fibrosis Panel. On the basis of additional data found in the literature, 4 other genes and the polymorphism rs35705950 of the *MUC5B* promoter were added in the custom panel for a total of 28 genes plus the *MUC5B* SNP: *ABCA3*, *AP3B1*, *ASAH1*, *COPA*, *CSF2RA*, *CSF2RB*, *DKC1*, *FAM111B*, *FARSB*, *FOXF1*, *GBA*, *HPS1*, *HPS4*, *ITGA3*, *MUC5B*, *NKX2-1*, *PARN*, *RTEL1*, *SFTPA1*, *SFTPA2*, *SFTPB*, *SFTPC*, *SLC34A2*, *SLC7A7*, *SMPD1*, *TMEM173*, *TERC*, *TERT*, *TERF1*. The panel probes were designed and synthetized with the support of the Integrated DNA Technology (IDT) company (https://eu.idtdna.com/site/order/ngs, accessed 23 September 2021), and the non-coding *MUC5B* promoter probes were added as Ultramers DNA Oligos for spike-in (Tema Ricerca, Bologna, Italy).

### 2.6. NGS

DNA library preparation was carried out using the Illumina DNA prep with enrichment kit and the custom-designed targeted sequencing panel (IDT) described above and loaded onto the Illumina MiSeq platform (Illumina, San Diego, CA, USA), with paired-end sequencing (300 cycles). Raw sequencing data were processed using an internal SNP and InDels calling pipeline [16], based on GATK best practices. Briefly, raw reads in Fastq format were trimmed with Fastp [17] and aligned to the reference genome hg38 using BWA-MEM (bio-bwa.sourceforge.net v.0.7.17-r1188). PCR duplicates were identified and marked using SAMtools (https://www.htslib.org/, accessed 12 July 2023). Alignment quality and coverage statistics were collected with SAMtools and GATK Depth of Coverage. Across all samples, our custom panel achieved a mean coverage of 140×, with an average of 99% of targeted positions covered at >20×. Per-gene coverage statistics are provided in Supplementary File S2. Variants were called and filtered by quality with GATK HaplotypeCaller and variant quality score recalibration (VQSR) and then annotated with Ensembl Variant Effect Predictor (www.ensembl.org/info/docs/tools/vep/index.html, accessed 23 September 2021). Candidate disease-causing variants were defined as variants with potential to alter the protein product (missense, nonsense, small insertion/deletions, and splicing-affecting variants) with allele frequency lower than 0.05 and not seen in homozygous state in gnomAD database. Variants were classified according to the guidelines described by the American College of Medical Genetics and Genomics (ACMG) [18].

### 2.7. Fibroblast Generation and Culture

The skin punch biopsy was obtained via standard procedure using a circular blade, yielding approximately 3 to 4 mm cylindrical core of tissue sample. The tissue was transferred in a culture dish and washed twice with Roswell Park Memorial Institute (RPMI) 1640 medium (Euroclone, Milano, Italy) and then once with AmnioMedPlus medium (Euroclone, Milano, Italy). The biopsy was dissected into evenly sized pieces using a sterile disposable scalpel and transferred into a sterile flask; the excess of medium was removed, leaving only a film of media coating the bottom of the well. The flask was then incubated at 37 °C in the presence of 5% CO_2_ for 24–48 h until fibroblasts attached; then, a small quantity of AmnioMedPlus was added to the culture, which was monitored daily until fibroblasts were confluent. Control human fibroblasts were purchased from ATCC (Manassas, VA, USA) and cultured following manufacturer’s instructions.

### 2.8. Reactive Oxygen Species (ROS) Detection on Cultured Fibroblasts

To detect the content of reactive oxygen species (ROS), the fibroblasts were cultured in 96-well plates (OptiPlate Black; PerkinElmer, Inc., Shelton, CT, USA) following manufacturer instructions and incubated with 10 µM of H_2_DCFDA (2′,7′-dichlorodihydrofluorescein diacetate; Thermo Fisher Scientific, Inc., Waltham, MA, USA), dissolved in the culture medium, for 30 min. As a positive control, cells were treated for 30 min with 100 µM of tert-butyl hydroperoxide (TBH) dissolved in culture medium. The cells were then washed with a Krebs–Ringer modified buffer solution containing 135 mM of NaCl, 5 mM of KCl, 1 mM of MgSO_4_, 0.4 mM of K_2_HPO_4_, 5 mM of glucose, and 20 mM of HEPES supplemented with 1 mM of CaCl_2_, pH 7.4, and the fluorescence emission from each well was measured (λ_(ex) 485 nm; λ_(em) 535 nm) using a multi-plate reader (EnSpire; PerkinElmer, Inc., Shelton, CT, USA). Data are normalized according to protein content as determined by the Lowry method.

## 3. Results

Genetic counseling was offered to 37 patients (26 males and 11 females; average age at the time of diagnosis, 69 ± 11; average age at the time of sampling, 73 ± 11 years) affected by familial forms of IPF, belonging to 31 families. The demographic features of the enrolled individuals are reported in Table 1.

### 3.1. CGH-SNP Array

Out of 37 patients analyzed, 20 (54.1%) were negative, and 17 (45.9%) were positive in the CGH-SNP array analysis. Among the 20 patients classified as negative for CNV, five patients showed at least one ROH stretch (SP1042(sr1041), SP1043, SP1073, SP1074, SP1189), and five cases, including two brothers (SP1070(frSP1071), SP1071(frSP1070)), showed a mosaic loss of Y chromosome (SP1068, SP1198, SP1313). Out of the positive cases, 14 duplications and 3 deletions were identified. Results are reported in Table 2.

Although patients show different CNVs, based on the gene content of the rearrangements identified, results can be grouped according to the mechanism potentially involved in IPF.

1. Mitochondria, mitophagy, ROS

Patients SP1010, SP1217, and SP1218 (these latter are two sisters) show a partial duplication of *PRKN* (MIM* 602544), whereas patient SP1066 shows a partial deletion of *GBE1* (MIM* 607839). Both genes are involved in ROS production, which is a predisposing factor of IPF [5]. In greater detail, alterations of *PRKN* have been reported as a cause of increased oxidative stress, altered mitophagy, and accumulation of dysmorphic mitochondria [19]. A reduction in *PRKN* is observed in the myofibroblasts of IPF lungs [20], and knockdown of the gene leads to increased mitochondrial ROS production and cellular senescence in human bronchial epithelial cells [21].

The partial deletion of *GBE1* could contribute to IPF, since a reduced expression of this gene has been related to hypoxia and increased intracellular ROS [22].

Patient SP1113 shows a duplication of *RYR2*, a gene that, in addition to its role in cardiomyopathies, has recently been associated with pulmonary hypertension, a documented complication of pulmonary fibrosis [23]. In this specific case, a direct correlation between *RYR2* and IPF is not reported in the literature but could only be inferred, since the mechanism of action relates to the increase in ROS.

2. NF-κb pathway

Patient SP1019 shows a complete duplication of *EDA2R* (also known as *XEDAR*, MIM* 300276), belonging to the tumor necrosis factor receptor (TNFR) superfamily. Patient SP1128 (brother of SP1229) shows a partial duplication of *IKBKG* (MIM* 300248) located in Xq28, a gene encoding for the regulatory subunit of the inhibitor of kappaB (IkB) IKK complex. The complete duplication of *EDA2R* may cause an overexpression of the gene which, in turn, is reported to induce NF-κB and inflammatory cascade through IKK complex-mediated phosphorylation and IκBα degradation [24]. Likewise, the *IKBKG* gene is required for the activation of the NF-kB pathway. Since both *EDA2R* [25] and *IKBKG* activate the NF-κB pathway, and NF-κB activation is involved in inflammatory phenomena and in the etiopathogenesis of IPF [26], these CNVs may be related to the etiopathogenesis of the disease. In addition to encoding genes, miRNA have also been described in association to IPF [27]. Patient SP1076 shows a 126 kb microduplication in Xq21.33 containing the miRNA MIR548M. Upregulation of MIR548M can contribute to the downregulation of *PTEN* [28], a gene whose reduced expression in IPF produces an increased expression of collagen and, through activation of the NF-kB pathway, senescence of epithelial cells [29].

3. Epithelial–Mesenchymal Transition (EMT)

The CNVs identified in patient SP1044, SP1075, and SP1101 may confer susceptibility to IPF in view of the gene content involved in multiple processes, mainly EMT. In patient SP1044, we detected a microduplication of the *NR2F2* gene (also known as *COUP-TFII*, MIM* 107773). The encoded protein promotes the transition from epithelial to mesenchymal cells [30] in the liver, but the gene is also expressed in the lung, at the level of the vascular endothelium of the alveolar septa [31]. *NR2F2* also increases glycolysis of myofibroblasts causing fibrosis [32]. The nr2f2 knockout mouse shows reduced glycolysis and reduced collagen 1 levels in fibroblasts, suggesting *NR2F2* targeting as a novel therapeutic approach to mitigate fibrosis in chronic kidney disease and, potentially, fibrosis in other organs [32]. In patient SP1075, we detected a partial duplication of *EXOC4* (also known as *SEC8*; MIM* 608185). *EXOC4* interacts with actin cytoskeleton remodeling and vesicle transport mechanisms. The protein is a component of the exocytosis complex, which is also essential for the biogenesis of the surface polarity of epithelial cells [33]. *EXOC4* regulates N-cadherin expression by controlling *SMAD3* and *SMAD4* expression at the basal transcriptional level, thus modulating cell migration and adhesion. Alterations in *EXOC4* expression levels act on the EMT process through the regulation of N-cadherin [34]. It should also be noted that Exoc4 is involved in tumor progression, acting on the proliferation and secretion of matrix metalloproteinases (MMPs) [35], which are known to be pathologically accumulated in fibrosis. Among the three genes (*EXOC3*, *AHRR*, *SLC9A3*) contained in the 115 kb duplication identified in case SP1101, *EXOC3* (also known as *SEC6*, MIM* 608186) belongs to the same complex as *EXOC4*, and it is involved in multiple cellular functions, including cell migration and suppression of apoptosis [36], epithelial cell polarity, NF-κB signaling, genome stability [37], and EMT. Since *AHRR* (MIM* 606517) is involved in apoptosis [38], and its overexpression leads to an increased expression of inflammatory genes and activates the NF-κB pathway [39], both genes may contribute to the development of pulmonary fibrosis.

4. Senescence, Endoplasmic reticulum (ER) stress

Patient SP1063 shows a 331 kb duplication in 11q22.3 containing *ALKBH8* (MIM * 613306), a gene which controls the translation of selenoproteins [40], which are metabolizing enzymes essential for the maintenance of the glutathione redox cycle (GSH) and involved in various biological processes such as epigenetic phenomena, oxidative stress, senescence, apoptosis, and cell growth [41]. *ALKBH8* deficiency leads to senescence and promotes mitochondrial reprogramming, as demonstrated by Alkbh8-deficient MEFs showing elevated markers of senescence [40]. In addition, an elevated expression of the gene is involved in cell growth and progression of some types of cancer (e.g., bladder cancer [42]). In consideration of the processes regulated by the gene (oxidative stress, senescence, apoptosis, and cell growth), a role of *ALKBH8* duplication cannot be excluded in etiopathogenesis of IPF.

5. Other different pathways possibly involved with the gene content of identified CNVs include cases SP1077 (*TOP3B* (MIM* 603582): DNA damage, R-loops processing, DNA recombination, cell aging, and genome stability), SP1076 (*SSR3*—also known as *TRAPG*- (MIM* 606213): endoplasmic reticulum stress, unfolded protein response (UPR), apoptosis [43]), SP1356 (*CELSR1* (MIM* 604523): required for normal lung branching morphogenesis, could play a role in developmental lung disease [44]), and SP1433 (*CSMD1* (MIM* 608397): fibroblast migration [45]).

In five cases (SP1070 (frSP1071), SP1071 (frSP1070), SP1068P1433, SP1198, SP1313), CGH-SNP analysis detected a mosaic Deletion of Y Chromosome (mLOY) as the only CNV variation. In a further case (SP1356), the Y loss was detected in addition to a CNV; therefore, this patient was included in the CGH-SNP array-positive group.

As discussed below, SP1068P1433 and SP1071 (frSP1070) show additional single-nucleotide variants: the first was positive for NGS (*PARN*), the latter was homozygote for canonical *MUC5B* polymorphism.

### 3.2. ROH Cases

Among patients negative to CNVs, two cases (SP1073, SP1074) showed ROH regions containing genes potentially implicated in pulmonary fibrosis. Patient SP1073 showed a 6.65 Mb ROH stretch at [hg38]8p23.2p23.1(6173230-12824573) containing *PINX1* (PIN2 (TERF1) interacting telomerase inhibitor; MIM* 606505), whereas SP1074 showed a 10.7 Mb ROH stretch at [hg38]15q21.1q21.3(45276664-55995585) containing *FGF7* (fibroblast growth factor 7; MIM* 148180). *PINX1* is involved in the maintenance of telomeres, whereas FGF7 is involved in fibroblast growth, two well-known processes involved in the etiopathogenesis of the disease. Although the ROH stretches identified in cases SP1073 and SP1074 contain genes potentially implicated in pulmonary fibrosis (*PINX1* and *FGF7*, respectively), without sequencing and identification of pathogenetic variants, any correlations between these ROHs and the disease are only hypothetical.

### 3.3. Target Gene Panel Analysis

“Pathogenic” or “likely pathogenic” variants were identified in 6/37 patients (16.2%), as indicated in Table 3.

**Figure 1:**
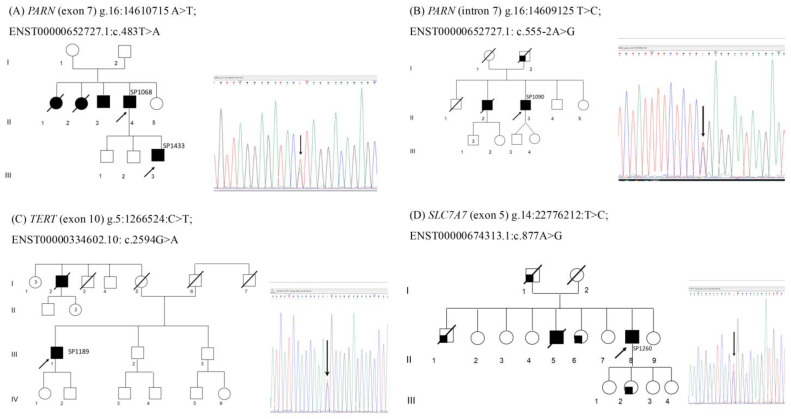
Genealogic trees of patients carrying the variants identified by NGS. A blackened (filled-in) shape indicates an individual affected by IPF, while a partial filling (e.g. (**B**) subject I.1, (**D**) subjects I.1, III.2) indicates an individual affected by respiratory diseases different from IPF. A line through any symbol signifies a deceased person (**A**) Patient SP1068 (subject II.4) and his son SP1433 (subject III.3) share an heterozygous nonsense variant in exon 7 of *PARN*, (**B**) Patient SP1090 (subject II.3) shows a splice acceptor variant in intron 7 of *PARN*, (**C**) Patient SP1189 (subject III.1) shows a heterozygous missense variant in exon 10 of *TERT*, (**D**) Patient SP1260(sr1075) (subject II.8) shows a heterozygous missense variant in exon 5 of *SLC7A7*

The genealogical trees of patients carrying these variants are shown in Figure 1.

The results from several individuals with known pathogenic variants were resequenced to validate the target gene panel. As an example, in patient SP1228 (fr1229) (previously tested by Sanger sequencing [46]), we confirmed the presence of the heterozygous missense variant in exon 2 of *TERT* (g.5:1294429 G>T; ENST00000334602.10: c.457C>A, ENSP00000334346.6: p.Leu153Met). The reported frequency of this variant in gnomAD is 6.281 × 10^−7^, and it is classified as likely pathogenic. This patient was included in the study both to validate the panel and to look for any additional variants/check the *MUC5B* genotype.

#### 3.3.1. PARN (MIM* 604212)

We identified a heterozygous nonsense variant in exon 7 of *PARN* (ENST00000652727.1: c.483T>A ENSP00000498650.1: p.Tyr161Ter) (Figure 1A, patient SP1068 subject II.4 and his son, SP1433 subject III.3). This variant, absent in several population databases (gnomADv4, deCAF, AllofUs), creates a premature nonsense codon, expected to lead to a loss of function of *PARN*, a gene where loss of function is a known mechanism of disease; therefore, it was classified as pathogenic. In patient SP1090 (Figure 1B, subject II.3), we identified a splice acceptor variant in intron 7 of *PARN* (g.16:14609125 T>C; ENST00000652727.1: c.555-2A>G) predicted to suppress the splicing acceptor site, which could result in exon skipping and an altered final transcript, with the insertion of a premature stop codon and a loss of function (SpliceAI tool [47], accessed 10 April 2025).

#### 3.3.2. TERT (MIM* 187270)

We identified a heterozygous missense variant in exon 10 of *TERT* (g.5:1266524:C>T; ENST00000334602.10: c.2594G>A, ENSP00000334346.6: p.Arg865His) (Figure 1C, patient SP1189 subject III.1). This variant causes a substitution of an arginine to histidine at amino acid position 865. This arginine is highly conserved and is part of the consensus sequence of motif C, one of seven motifs conserved in all reverse transcriptase proteins. This variant has already been described as causative of pulmonary fibrosis [48].

#### 3.3.3. SLC7A7 (MIM* 603593)

We identified a heterozygous missense variant in exon 5 *SLC7A7* (g.14: 22776212 T>C; ENST00000674313.1: c.877A>G, ENSP00000501493.1: p.Ser293Gly) (Figure 1D, patient SP1260(sr1075) subject II.8). This variant causes a substitution of a serine to glycine at amino acid position 293 and is classified as likely pathogenic. The additional *SLC7A7* intronic variant ENST00000674313.1: c.-42-53T>G identified is not predicted to impact splicing and, since *SLC7A7* is associated with fibrosis in a biallelic mode of inheritance, these results do not explain the disease in patient SP1260 (sr1075).

#### 3.3.4. MUC5B

The canonical rs35705950 SNP was identified in heterozygosity in 18 patients (see Table 3) and in homozygosity in 2 patients (SP1071, SP1388). A MUC5B SNP rs141420125 (RefSeqGene NG_031880.1:g.2067A>G SNV:11-1220131-A-G (GRCh38), gnomAD f = 0.003062) located 141 bp downstream from the canonical one, rs35705950 (RefSeqGene NG_031880.1:g.1927G>A SNV:11-1219991-G-A (GRCh38), gnomAD f = 0.07938), and not previously described in association with IPF, was identified in heterozygosity in patient SP1228 and in compound heterozygosity with the canonical SNP in two sisters (patients SP1041 and SP1042). Altogether, 23 patients out of 37 (62.2%) showed at least one MUC5B SNP (summarized in Table 4), whereas 15 patients were wild-type to both MUC5B SNPs. Out of the 23 patients positive to *MUC5B*, 12 were positive also to CGH-SNP array, and one was positive to CGH and NGS.

### 3.4. ROS Detection on Cultured Fibroblast

**Figure 2:**
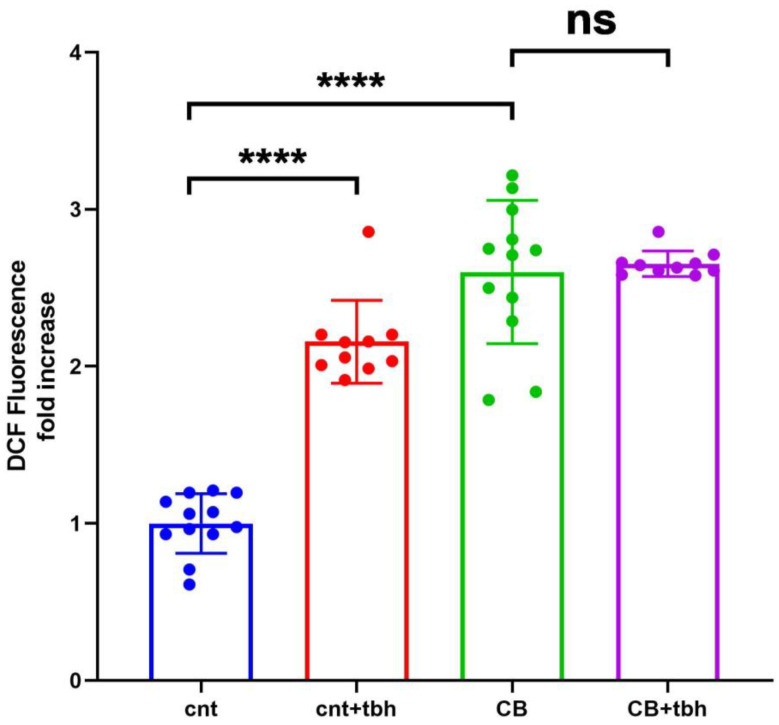
Analysis of ROS in patient’s fibroblasts. Endogenous and induced ROS evaluation showed a statistically significant increase (**** *p* < 0.0001) in patient’s fibroblasts (SP1066, CB, green box) vs. control fibroblasts (cnt. blue box). The patient’s fibroblasts have significantly higher basal oxidative stress, comparable to the one induced by Tert-butylhydroperoxide (TBH) in control samples (cnt + tbh, red box). Patient’s fibroblasts induced by TBH (CB+tbh, purple box) do not show a significant increase of ROS vs patients endogenous fibroblast (CB, green box) (ns = not significant).

DCFDA staining detected a significantly increased production, compared to control cells, of ROS in the SP1066 patient’s skin-derived fibroblasts carrying the partial deletion of the GBE1 gene (Figure 2).

## 4. Discussion

IPF is a condition characterized by a complex etiopathogenesis in which many different molecular mechanisms and biological processes come into play, and alterations of several genes involved in these processes can contribute to the development of the disease. NGS data reported in the literature have mainly focused on genes involved in the formation of pulmonary surfactant and in telomere maintenance, whereas the analysis of the genome via CGH + SNP array allows for broad-spectrum evaluation of structural alteration in all the other genes involved in this and in other biological processes.

In order to identify possible predisposing variants to the disease, in this study, we analyzed, both by an NGS custom panel and by CGH + SNP array, a small cohort of 37 selected Italian patients with familial forms of pulmonary fibrosis. Out of the 37 tested, 29 patients (29/37; 78.4%) were positive either in CNV or NGS/*MUC5B* analysis (Table 3). Among the eight patients (8/37; 21.6%) who did not show neither a CNV nor a SNV/*MUCB* SNP, three had a mosaic loss of Y chromosome (SP1070, SP1198, SP1313), and two had an ROH stretch (SP1073, SP1074), leaving only three patients (SP985, SP1069, SP1229) without any genetic predisposing factor (included in the genetic tests performed) to IPF.

As reported in the Results section, the CNVs identified by CGH+SNP contain genes implicated in different pathways which could be potentially involved in IPF, including the following: (i) mitochondria, mitophagy, ROS; (ii) NF-κb pathway; (iii) EMT; (iv) senescence, endoplasmic reticulum (ER) stress; (v) other pathways, including DNA damage and lung development pathways.

In relation to the mLOY cases, it is interesting to note that in the pair of siblings with this alteration (SP1070 and SP1071), the subject with a higher percentage of mosaicism (45% vs. 23%) shows a more severe form of disease that already requires oxygen supplementation. The loss of the Y chromosome in men, occurring usually in old age but described as early as at 20 years of age [49], is a phenomenon associated with an increased risk of pathologies, including an increased incidence of various tumors, among which lung cancer represents 4.5% [50]. The recent data of Wang D et al. [51] demonstrate that mLOY is greater in male patients with PF compared with non-PF patients. Single-cell transcriptomic analysis of lung tissue from patients with a variety of fibrotic diseases identified that mLOY in immune cells is associated with fibrotic diseases, and there appeared to be an association with increased fibrosis within samples. It is also interesting to note that the pseudoautosomal region of the Y chromosome contains *CSFR2A*, a gene which causes pulmonary fibrosis in a recessive mode of inheritance. The loss of the Y chromosome could therefore represent an additional risk factor and could increase susceptibility to the disease because it corresponds to the loss of a gene implicated in pulmonary fibrosis, which, as described, is a disease that occurs mainly in old age. In the three analyzed cases, we can consider, by virtue of the age and the presence of offspring for all patients, the deletion identified as a somatic rather than germinal alteration.

By NGS analysis, we detected novel and already-reported variants. A novel heterozygous nonsense variant in *PARN* (p.Tyr161Ter) was shared by patient SP1068 and his son SP1433; the latter was also positive in CGH array, which detected the partial duplication of *CSMD1* (NM_033225.6, exon 1), a gene predicted to be haploinsufficient. *CSMD1* expression is reduced in human hypertrophic tissue, whereas in vitro studies demonstrated that its knockdown resulted in enhanced migration and fibronectin1 (FN1) secretion in fibroblasts [45]. Interestingly, SP1433 developed IPF at a younger age compared to his father, thus suggesting an additive effect of multiple genetic factors on the age of onset of the disease. The same possible “additive” effect could be hypothesized for SP1228 (brother of 1229), who tested positive for both CGH and NGS and developed the disease at a younger age.

By the application of a custom panel, we showed that most patients (23/37, 62.2%) were positive either to the canonical *MUC5B* SNP rs35705950, or to a novel *MUC5B* SNP, rs141420125, that was never described in association with IPF 

The data in the literature state that genetic alterations are identifiable in about 25% of familial forms of pulmonary fibrosis [52], while this percentage reaches 44% in the case of neonatal respiratory diseases [53]. However, it is important to underline that at present, the literature data are mainly focused on the analysis of single-nucleotide variants (NGS panels), while there is no evaluation of the genomic rearrangements that could contribute to the onset of the phenotype. Although single-nucleotide variants represent most of the causative variants described in association with IPF, deletions and duplications can predispose to disease, as happens in a number of genetic pathologies. Furthermore, in consideration of the multiple mechanisms underlying the etiopathogenesis of pulmonary fibrosis, it is possible to hypothesize that both CNVs and SNVs of genes involved in these processes may contribute to the onset of the phenotype in some patients. The CNVs identified in this work, although not related to genes already identified as causative of IPF, include genes potentially involved in mechanisms that have been shown to play a role in the pathogenesis of the disease, such as oxidative stress, epithelial–mesenchyme differentiation, altered mitophagy, and inflammatory processes regulated by the NF-κB pathway. These rearrangements are not described as population polymorphisms in the available online databases (Database of Genomic Variants (DGV) https://dgv.tcag.ca/dgv/app/home, DatabasE of genomiC varIation and Phenotype in Humans using Ensembl Resources (DECIPHER), https://www.deciphergenomics.org/, ClinVar https://www.ncbi.nlm.nih.gov/clinvar/, accessed 10 April 2025) and have not been identified in the in-house database of patients followed for other clinical indications evaluated by our Medical Genetics Service using CGH + SNP array, thus reducing the likelihood of having identified neutral variants.

In order to confirm the significance and the potential role of the rearrangements identified in the etiopathogenesis of pulmonary fibrosis, expression studies will be necessary to confirm the increased (in the case of duplication) or reduced (in the case of partial deletion or duplication of the gene interrupting the sequence) expression of the genes contained within the identified rearrangements. In some cases, such as the partial duplication of *PRKN*, there are studies in the literature which show a reduced expression in the case of duplications involving only some exons of the gene (as is our case). In the case of *EDA2R*, it has been shown that overexpression of the gene, induced by cellular transfection, induces the NF-κB pathway [25]; however, it remains to be demonstrated, although it is probable, that the complete gene duplication observed in patient SP1019 induces overexpression. Following the expression studies, functional studies will be necessary to verify that the hypo/overexpression interferes, as expected, in the metabolic pathways mentioned above, such as, for example, the evaluation of ROS levels in skin fibroblasts as we did for case SP1066 or, if available, lung epithelial cells obtained by biopsy. The evaluation of oxidative stress is particularly interesting, as it plays a central role in the development and progression of IPF, and antioxidant therapies have been proposed for many years [54,55].

Actually, the search for genetic alterations for pulmonary fibrosis has not yet taken into account the alterations in the number of copies. From the identification of genes in single families by cloning, we have moved on to genome-wide association studies that have made it possible to identify SNVs in potentially related genes.

The use of array-CGH for the search of susceptibility CNVs, as recently performed for chronic obstructive bronchopathy [56], has not yet been described for IPF, whereas CNVs of susceptibility have been reported in single genes predisposing to pulmonary fibrosis (e.g., *FCGR3B* in [57]).

## 5. Conclusions

Although the recent literature has not reported the use of the CGH+SNP array test in the clinical–genetic diagnosis of IPF, our data obtained in a small series of patients with familial forms of pulmonary fibrosis, suggest that, CNVs potentially related to the disease (if confirmed by expression and functional studies) are found in a high percentage of cases (45.9%). Interestingly, in two patients who developed the disease at a younger age, a positivity in the CGH array was also associated with a positivity in NGS, suggesting the hypothesis of a possible additive effect of multiple genetic factors on the age of onset of the disease, which will require further confirmation. We are aware that our study shows many limitations, such as the limited sample size and the single ethnicity of patients analyzed (only Italians), which could introduce a population bias. However, the detection rate of duplications and deletions identified suggest that (as happens for the majority of genetic disease) structural rearrangement may play a substantial role in the etiopathogenesis of diseases, and the detection rate of duplication/deletion could potentially be even higher if a platform with higher density/resolution were used (i.e., XON array, which could detect deletion or duplication of a single exon). Nevertheless, as a starting point, application of the CGH + SNP array to selected patients can at least be evaluated and proposed in the diagnostic path of these pathologies. On the research side, further functional studies may also clarify the role, if any, of the novel *MUC5B* polymorphism.

## Figures and Tables

**Table 1:** Clinical data of patients enrolled in the study.

Demographics Features	*n* = 37
Gender (M/F)	26/11
Age (mean + SD)	69 + 11
**Diagnosis, *n* (%)**	
IPF	29 (78%)
CPFE (with UIP pattern)	5 (14%)
Fibrotic HP	2 (5%)
Other fibrosing-ILD	1 (3%)
**Number of family members affected, *n* (%)**	
0	3 (8%)
1	16 (43%)
2	8 (22%)
3	3 (8%)
4 or more	7 (19%)
**First degree of affected relative, *n* (%)**	
Sibling	25 (68%)
Parent	10 (30%)
Son/daughter	1 (3%)
**Second degree of affected relative, *n* (%)**	
Aunt/uncle	9 (24%)
**Smoking history, *n* (%)**	
Never smoker	15 (41%)
Current/former smoker	22 (59%)
**HRCT pattern, *n* (%)**	
UIP definite/UIP probable	21 (57%)
Indeterminate UIP	11 (30%)
CPFE	5 (13%)

**Table 2:** CGH-SNP results. Information and CGH-SNP array results relating to the patients analyzed. Each patient is indicated with an alphanumeric code SPXXX (first column), gender of the patient (M = male, F = female; second column), age at the time of sampling (third column), CGH results positive (+) or negative (−) (fourth column), CGH result ISCN 2020, Hg38 (fifth column), CNV/gene potentially related to IPF (sixth column), ROH: run of homozygosity (seventh column).

Patient Code	Sex	Age	CGH	CGH-SNP Results [hg38]	CNV/Gene Potentially Related to FP	Run of Homozygosity (ROH)
SP985	M	77.1	−			
SP1010	F	80.2	+	6q26(162233059-162579288)x3 VUS	Gain6q26 (346kb) partial dup *PRKN*	
SP1019	M	68.8	+	Xq12(66548368-66706421)x2 VUS	Gain Xq12 (158kb) partial dup *EDA2R*	
SP1028	M	70.3	−			
SP1041(sr1042)	F	83.0	−			
SP1042(sr1041)	F	81.8	−	ROH 3 (8.7 Mb)		3q22.1q22.3(129671286-138395712)x2 hmz (8.7Mb)
SP1043	M	67.1	−	ROH 2,7,12 (58 Mb)		2q22.1q24.3(140295318-167683159)x2 hmz (27Mb); 2q36.3q37.3(227813126-239155675)x2 hmz (11Mb); 7p15.3p14.3(23069414-28893706)x2 hmz (5.8Mb); 12q21.2q21.33(75496872-89230429)x2 hmz (13.7Mb)
SP1044	M	68.6	+	15q26(96330212-96335401)x3 VUS	Gain 15q26 (5.2kb) partial dup *NR2F2*	8q24.22q24.3(131543882-139009356)x2 hmz (7.4Mb)
SP1063	M	75.3	+	11q22.3(107356495-107688074)x3 VUS	Gain 11q22.3 (331 kb) dup *ALKBH8*	
SP1066	F	54.2	+	3p12.2(81494599-81700597)x1 VUS, 13q21.33(69771139-70889061)x3 (1.12 Mb) VUS	Loss 3p12.2 (205 kb) partial del *GBE1*	
SP1067	F	79.6	−			
SP1068P1433	M	72.2	−	loss Y (50% mosaic)	Loss Y mosaic	
SP1069	M	75.7	−			
SP1070(frSP1071)	M	73.3	−	loss Y (45% mosaic)	Loss Y mosaic	
SP1071(frSP1070)	M	77.8	−	loss Y (24% mosaic)	Loss Y mosaic	
SP1072	M	76.8	−			
SP1073	F	83.0	−	ROH 8, 13 (13.6Mb)		8p23.2p23.1(6173230-12824573)x2 hmz (6.7 Mb); 13q31.1q31.3(83118709-90076330)x2 hmz (7Mb)
SP1074	M	68.8	−	ROH 15 (10.7Mb)		15q21.1q21.3(45276664-55995585)x2 hmz (10.7Mb)
SP1075(fr1260)	M	76.6	+	7q33(133930262-134321394)x3 VUS	Gain 7q33 (391 kb) partial dup *EXOC4*	
SP1076	M	85.8	+	3q25.31(156188551-156696052)x3 VUS	Gain 3q25.31 (507kb) dup *SSR3*	
SP1077	M	73.8	+	22q11.22(21959009-22202339)x1 VUS	Loss 22q11.22 (243 kb) partial del *TOP3B*	
SP1090	M	75.0	−			
SP1101	F	77.2	+	5p15.33(432377-548016)x3 VUS	Gain 5p15.33 (115 kb) partial dup *AHRR*, dup *EXOC3*	7p13p12.1(45370510-52312470)x2 hmz (6.9Mb)
SP1113	M	82.9	+	1q43(237356321-237827238)x3 VUS	Gain 1q43 (470 kb) partial dup *RYR2*	
SP1189	M	64.4	−	ROH 1 (18Mb)		1q24.2q31.1(168657469-186623952)x2 hmz (18Mb)
SP1190	F	69.0	+	Xq21.33(95025484-95151628)x3; ROH 5,10,13 (78.6 Mb)	GainX (126 kb) miRNA *MIR548M*	5q33.2q35.1(156128234-171751925)x2 hmz (15.6Mb); 10q21.1q21.3(56076047-67561901)x2 hmz (11.5Mb); 13q14.11q31.3(40483478-91981823)x2 hmz (51.5Mb)
SP1198	M	70.2	−	Loss Y mosaic (14% mosaic)	Loss Y mosaic	
SP1217(fr1218)	M	89.6	+	6q26(162327256-162629371)x3 VUS	Gain 6q26 (302 kb) partial dup *PRKN*	
SP1218(sr1217)	F	80.4	+	6q26(162327256-162629371)x3 VUS	Gain 6q26 (302 kb) partial dup *PRKN*	
SP1228(fr1229)	M	50.8	+	Xq28(154503086-154555424)x3 VUS	Gain Xq28 (52kb) partial dup *IKBKG*	
SP1229(fr1228)	M	60.0	−			
SP1260(sr1075)	F	90.0	−			
SP1313	M	94.0	−	Loss Y (45% mosaic)	Loss Y mosaic	
SP1356	M	66.2	+	22q13.31(46501744-46791748)x3 VUS + loss Y (30% mosaic)	Gain 22q13.31 (290 kb) partial dup *CELSR1* + loss Y mosaic	
SP1388	M	57.4	+	8p11.21p11.1(43086764-43883660)x3 LB	Gain 8p11.21p11.1 (796 kb) dup *HGSNAT* (pulmonary hypertension)	
SP1433(fg1068)	M	41.4	+	8p23.2(4867587-5099066)x1 VUS	Loss 8p23.2 (231 kb) partial del *CSMD1*	
BO-39514	F	75.4	−			

VUS = variant of uncertain significance, LB = likely benign variant.

**Table 3:** NGS results relating to the patients analyzed. Each patient is indicated with an alphanumeric code SPXXX (first column); the gender of each (M = male, F = female) is reported in the second column. NGS results (columns from 4 to 8): only genes with pathogenic (P) or likely pathogenic (LP) variants are included in the table. MUC5B results (column 9) report the specific *MUC5B* SNP identified in each patient; het = heterozygote, hom = homozygote. Summary of genetics tests (columns 10–12): test positive (+), test negative (−).

			NGS Results					MUC5B Results	Summary of Genetic Results		
Patient Code	Sex	Gene with P/LP Variant	NGS Variant (P/LP)	Classification	N° Variants	Mono (M)/Biallelic (B) Gene	Compatibility of the Identified Variant/Mode of Inheritance	MUC5B SNP Canonical = rs35705950; Novel = rs141420125	CGH	MUC5B	NGS
SP985	M								−	−	−
SP1010	F							Canonical het	+	+	−
SP1019	M							Canonical het	+	+	−
SP1028	M							Canonical het	−	+	−
SP1041 (sr1042)	F							Novel het + Canonical het	−	+	−
SP1042 (sr1041)	F							Novel het + Canonical het	−	+	−
SP1043	M							Canonical het	−	+	−
SP1044	M							Canonical het	+	+	−
SP1063	M							Canonical het	+	+	−
SP1066	F								+	−	−
SP1067	F							Canonical het	−	+	−
SP1068 P1433	M	*PARN*	ENST00000652727.1:c.483T>A ENSP00000498650.1:p.Tyr161Ter	P	1	M	Yes		−	−	+
SP1069	M								−	−	−
SP1070 (frSP1071)	M								−	−	−
SP1071 (frSP1070)	M							Canonical hom	−	+	−
SP1072	M							Canonical het	−	+	−
SP1073	F								−	−	−
SP1074	M								−	−	−
SP1075 (fr1260)	M							Canonical het	+	+	−
SP1076	M							Canonical het	+	+	−
SP1077	M							Canonical het	+	+	−
SP1090	M	*PARN*	ENST00000652727.1:c.555-2A>G splice_acceptor_variant	P	1	M	Yes		−	−	+
SP1101	F							Canonical het	+	+	−
SP1113	M								+	−	−
SP1189	M	*TERT*	ENST00000334602.10:c.2594G>A ENSP00000334346.6:p.Arg865His	P	1	M	Yes	Canonical het	−	+	+
SP1190	F							Canonical het	+	+	−
SP1198	M								−	−	−
SP1217 (fr1218)	M							Canonical het	+	+	−
SP1218 (sr1217)	F							Canonical het	+	+	−
SP1228 (fr1229)	M	*TERT, RTEL1*	TERT ENST00000334602.10:c.457C>A ENSP00000334346.6:p.Leu153Met, RTEL1 ENST00000508582.6:c.3662G>C ENSP00000424307.2:p.Gly1221Ala		1 + 1	M	Yes	Novel het	+	+	+
SP1229 (fr1228)	M								−	−	−
SP1260 (sr1075)	F	*SLC7A7*	ENST00000674313.1:c.877A>G ENSP00000501493.1:p.Ser293Gly + intron variant ENST00000674313.1:c.-42-53T>G	LP	1 + 1	B	?	Canonical het	−	+	+
SP1313	M								−	−	−
SP1356	M								+	−	−
SP1388	M							Canonical hom	+	+	−
SP1433 (fg1068)	M	*PARN*	ENST00000652727.1:c.483T>A ENSP00000498650.1:p.Tyr161Ter	P	1	M	Yes		+	−	+
BO-39514	F							Canonical het	−	+	−

We reported a ? in case SP1260 because *SLC7A7* is a recessive gene, and we identified 2 variants, but one is an intronic variant (therefore a VUS and we cannot confirm its pathogenetic role) so we cannot be sure that the combination of these 2 variants can cause the disease in a biallelic mode of inheritance.

**Table 4:** Summary of the genetic analysis.

CGH (+)	MUC5B (+)	NGS (+)
17/37	23/37	5/37
45.9%	62.2%	13.5%

## Data Availability

The raw data supporting the conclusions of this article will be made available by the authors on request.

## Supplementary Materials

The following supporting information can be downloaded at: https://www.mdpi.com/article/10.3390/biomedicines14010138/s1, Supplementary File S1: Reports the primers sequences, Tm and amplicon length of the primers designed by the Primer3 tool (https://primer3.ut.ee/, accessed 23 September 2021); Supplementary File S2: reports per-gene coverage statistics.

## Abbreviations

The following abbreviations are used in this manuscript:

CGH-SNP	Comparative genomic hybridization + single-nucleotide polymorphism
CN	Copy number
CNV	Copy number variations
CPFE	Combined pulmonary fibrosis and emphysema
EMT	Epithelial–mesenchymal transition
ER	Endoplasmic reticulum
HP	Hypersensitivity pneumonitis
HRCT	High-resolution computed tomography
IPF	Idiopathic pulmonary fibrosis
NGS	Next-generation sequencing
RPMI 1640	Roswell Park Memorial Institute Medium 1640
ROH	Run of homozygosity
ROS	Reactive oxygen species
RT-qPCR	Quantitative real-time polymerase chain reaction
UIP	Usual interstitial pneumonia
