# Supplementary material for: Idiopathic Pulmonary Fibrosis: Analysis of Predisposing Variants in Patients with Familial Forms
Source: Biomedicines. 2026 Jan 9;14(1):138. doi: 10.3390/biomedicines14010138 (PMC12838683; doi:10.3390/biomedicines14010138)
Supplement: Supplementary file 1 [file biomedicines-14-00138-s001.zip › Supplementary file S1 Real Time primers proof accepted rev.pdf]

Supplementary file S1. Features of the primers used for Real time PCR

Primers name	Sequence	T_m	Amplicon length in base pair
ALKBH8_ex2_FOR	GGACAGCAACCATCAAAGTAATT	57.61	115
ALKBH8_ex2_REV	GGATACTGTCTCAATGCCTTCA	57.86	
ALKBH8_int3_FOR	tcatgttcacaccggctact	59.03	130
ALKBH8_int3_REV	gctgaaaggcacccaacata	58.46	
ALKBH8_int5-ex6_FOR	tgcttgtgggtttattaatggac	57.35	161
ALKBH8_int5-ex6_REV	AGCTTTCACAAATGTCAGGAAG	57.16	
EDA2R_intr1_FOR	gcttggtctctttgcctcag	58.84	171
EDA2R_intr1_REV	gacgaggaagccaacacaaa	58.69	
EDA2R_intr2_ex3_FOR	gatcccatctccaagctcctat	58.82	182
EDA2R_intr2_ex3_REV	TCTGAACACGATTGATGACAGC	59.00	
EDA2R_intr3_ex4_FOR	ttgatcaccctgccattcct	59.00	186
EDA2R_intr3_ex4_REV	taccctatcttgctgccacc	59.16	
EDA2R_intr5_FOR	cacatgaatcaggaacaaggct	58.91	190
EDA2R_intr5_REV	ttctaaactctcagggttggct	59.02	
EDA2R_ex6_FOR	gGAGGTTTGCTGCAGTTTGA	58.97	199
EDA2R_ex6_REV	AGGAGGCCATGGTAAAGGAC	59.08	
EDA2R_ex7_FOR	AGGGAGAAGGGCTGAAGTTC	59.01	199
EDA2R_ex7_REV	GAACGGTGGTGAGAAAGCTG	59.13	
EDA2R_upstream_FOR	GCCAGTGAAGAAGTGTGTGG	59.05	190
EDA2R_upstream_REV	CTCAGCCTCAGATGCACAAC	58.91	
EDA2R_downstream_FOR	GGGCCTCATACGCTATCCAA	59.31	192
EDA2R_downstream_REV	TGACATCTCTTGCCTGGGTT	58.93	
EXOC3_int1_FOR	atcttcgcaatgtccatgtctc	58.80	90
EXOC3_int1_REV	accttcgtaacacaaacatgca	59.05	
EXOC3_int3_FOR	cagctttaggaacacatcagca	58.92	105
EXOC3_int3_REV	tggttaactgtggtcatttgca	58.71	
EXOC3_int6-ex7_FOR	cagatggaggtttcatataacagca	58.94	133
EXOC3_int6-ex7_REV	TCTTCACTTATCTGAGCAGCAAC	58.75	
EXOC4_int13_FOR	gagagcagtatagacgccatcc	60.09	90
EXOC4_int13_REV	gtgactccaatgattgtgcctt	59.18	
EXOC4_ex-int15_FOR	TCAGTGAACTTGCCAAATCGTT	59.05	110
EXOC4_ex-int15_REV	gcattcaaattgcaaggtcctg	59.00	
EXOC4_int16-ex17_FOR	atgcctctaatctgtcatccgt	59.03	94
EXOC4_int16-ex17_REV	GCACCATTAATGAGGATGCAGG	59.70	
EXOC4_upstream_FOR	TCAATTCCTCAACATGGTGTGC	59.44	114
EXOC4_upstream_REV	CCATCAGGCTCAAACGTTACTT	58.93	
EXOC4_downstream_FOR	GGACTATGCTCGGTGTTAGGAT	59.37	103
EXOC4_downstream_REV	ATTCTTCACCGTACACACCTCT	59.10	
GBE1_int3-ex4_FOR	cctttcctacttgtagcagtgc	59.00	131
GBE1_int3-ex4_REV	ACGATACAAGATCTCTCCGCTT	59.05	

GBE1_ex7-int6_FOR	cggctgtttcctctaaatgtga	58.67	92
GBE1_ex7-int6_REV	CCAGTTCTTGTAGCTCTTCAGG	58.41	
GBE1_ex13_FOR	GACCTTCTTCGCTACAAGTTCC	59.01	106
GBE1_ex13_REV	tccagagtgaagagcttacCTG	59.17	
GBE1_upstream_FOR	ACCTTAGCTGATAGAACTGACG	57.36	90
GBE1_upstream_REV	CTTAATAAACGATGAACTGGGTCTG	57.99	
GBE1_downstream_FOR	TAACATGGAATCAGGAGGAGCA	58.89	112
GBE1_downstream_REV	CTAAGTGGCTGCATTCCTGAG	58.71	
NR2F2_int1_ex2_FOR1	ctgaggctggtcattaactgtg	58.99	164
NR2F2_int1_ex2_REV1	CGCAACAGCAGGGAAATATATC	57.59	
NR2F2_int1_ex2_FOR2	TGGACCACATACGGATCTTCC	59.24	206
NR2F2_int1_ex2_REV2	tcaaggagcagactgggttt	58.86	
NR2F2_upstream_FOR	CTTGGCTTCATTCTGTGGCA	58.75	171
NR2F2_upstream_REV	ACCTCAGCCCTCACTCTTTC	59.02	
NR2F2_downstream_FOR	GGTTGCATGTGTGTTCTGGT	58.97	151
NR2F2_downstream_REV	CTTGGTAAAGGCGGTTCAGG	58.83	
PARK2_int1_ex2_FOR	cattcactgaagggctgcg	59.20	163
PARK2_int1_ex2_REV	TCAGAATCGACCTCCACTGG	58.81	
PARK2_int2_ex3_FOR	ccacggagggcaagttaaac	59.12	175
PARK2_int2_ex3_REV	GTCGCCTCCAGTTGCATTC	59.20	
PARK2_upstream_FOR	CCTGCTCGCTCTGAATTGTC	58.99	166
PARK2_upstream_REV	GATTGCTGTTGGGTTGTGGA	58.67	
PARK2_downstream_FOR	AGCTGGAGTATGTTCACTGGAA	59.09	100
PARK2_downstream_REV	CTTCTCACTGCTTCCTTTCCTG	58.93	
RYR2_int9-ex10_FOR	tgacagtccagacctgaatgat	58.82	128
RYR2_int9-ex10_REV	TGCTCTTCACCATGTTCTCCT	59.02	
RYR2_ex13_FOR	AGTAGATGGCATGGGAACATCT	59.85	125
RYR2_ex13_REV	TAGATCCCATTCTCACGGATTT	59.80	
RYR2_int17_FOR	actttgagtgaggtgtggtggt	60.89	121
RYR2_int17_REV	atgttggagtgcagtgaccata	60.44	
SSR3_ex1_FOR	ggctctacGTTCCCTGTTCT	59.10	163
SSR3_ex1_REV	GGATGGCAGACACGATGAAC	58.99	
SSR3_ex3-int2_FOR	acggtgtgtttctgagagagt	58.97	102
SSR3_ex3-int2_REV	AGCATCCTCCCTCTTCTGTG	58.80	
SSR3_ex4_FOR	ctgtgtctgtagAATCTTGTGGA	57.86	104
SSR3_ex4_REV	CAATGACCACGACCAGGAAC	58.84	
TOP3B_ex1_FOR	gagccacggaacctaagaac	58.28	99
TOP3B_ex1_REV	cctatttccgggtccagcc	59.85	
TOP3B_ex3_FOR	CCTCACACAAAGGGCTGAAC	59.05	117
TOP3B_ex3_REV	AATCCAGGGTCATCACGTGA	58.72	
TOP3B_int4_FOR	tcaacacagccagctcctta	58.94	146
TOP3B_int4_REV	cagcacaggttcacacgtac	59.14	
MIR548 up_FOR	gaccacagttcttctttcacct	58.19	107

MIR548 up_REV	tggtattatcctcgctttccca	58.96	
MIR548 down_FOR	cccaggagaggtctgtattaac	57.33	90
MIR548 down_REV	tctgaagagtagtgtgacagca	58.78	
IKBKG Int1_FOR	ctaccattcagcccactacact	59.50	110
IKBKG Int1_REV	tttgtggagggagatgtcagta	58.48	
IKBKG Int1(2)_FOR	gattcaggacccaggttacttg	58.39	93
IKBKG Int1(2)_REV	tcacacaaggagtagggtcag	58.75	
CELSR1-Int1_FOR	cttggagatgctctttcgtcag	59.07	137
CELSR1-Int1_REV	gcattatagggtgactcaggga	59.03	
CELSR1-Ex1_FOR	CATTCACTACAGCATCCTCAGC	59.13	118
CELSR1-Ex1_REV	CAGCGAGTATTTCTGGACATCC	58.87	
HGSNAT_Ex2_FOR	GATCAGGCTTTGCTACTCATCC	58.86	119
HGSNAT_Ex2(Int2)_REV	cccaaagcaaacagaactgagt	59.31	
HGSNAT_ Int9_FOR	ttcaggtgcagaagggtgtata	58.82	108
HGSNAT_ Int9_REV	tcgaagccacagtaatgtcttc	58.41	
CSMD1 up_FOR	cagcaaggaagggagatcaaag	58.98	90
CSMD1 up_REV	ggaacaatgtaatccggcaact	59.25	
CSMD1-Ex1_FOR	CCTCGGGTGATTATTTGGCT	57.65	90
CSMD1-Ex1_REV	TCATGTCTGCAGATACTCCACA	58.90	
FOXP2_FOR	TGCTAGAGGAGTGGGACAAGTA	65.3	98
FOXP2_REV	GAAGCAGGACTCTAAGTGCAGA	64.8	

Supplementary file S1 reports the features of the primers used for Real time PCR and designed by the Primer3 tool (https://primer3.ut.ee/). Primer's name includes the exon (ex)/intron (int) of the gene amplified (first column); nucleotide sequence of each primer, capital letters refer to exon, lowercase represent introns (second column); melting temperature Tm indicates the temperature at which 50% of the DNA strands dissociate into single strands (column 3); amplicon length in base pair (column 4)
