# Supplementary figures and images for: Idiopathic Pulmonary Fibrosis: Analysis of Predisposing Variants in Patients with Familial Forms
Source: Biomedicines. 2026 Jan 9;14(1):138. doi: 10.3390/biomedicines14010138 (PMC12838683; doi:10.3390/biomedicines14010138)

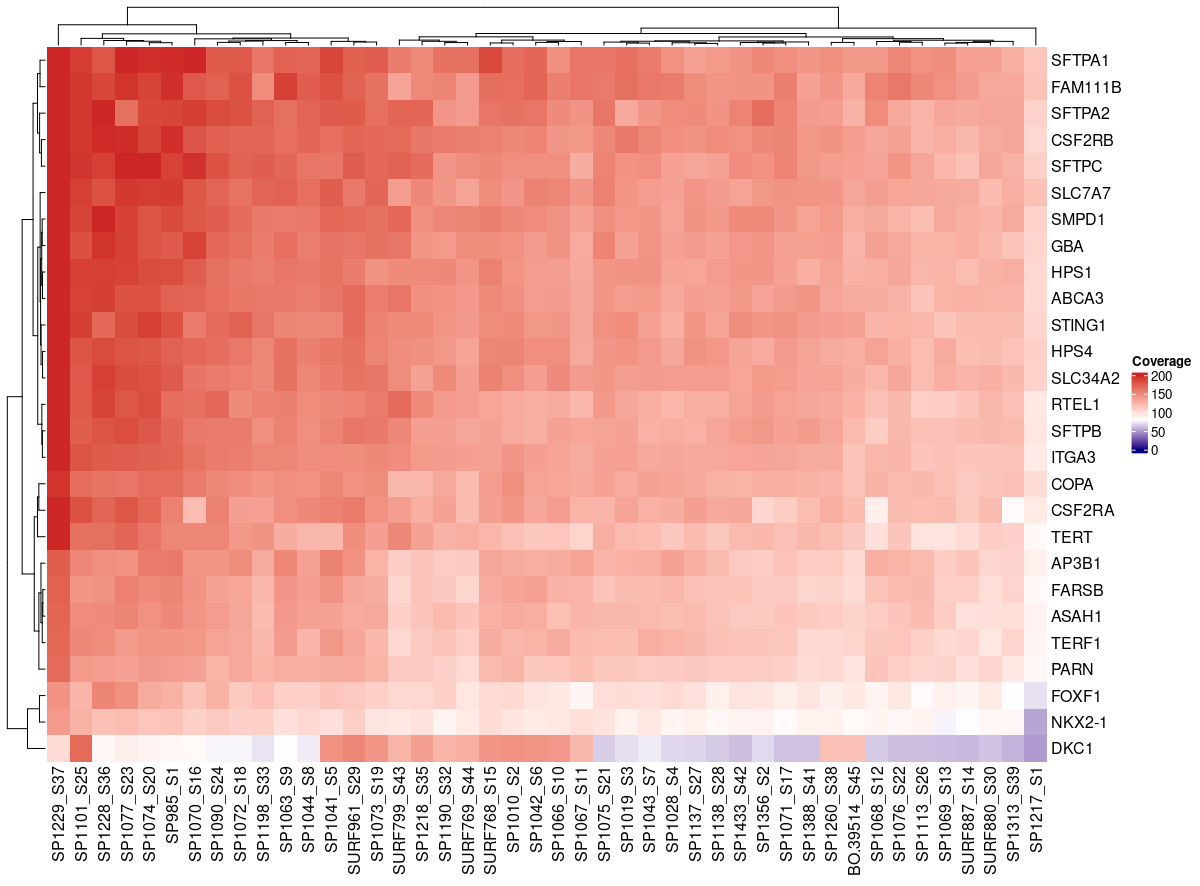

Supplement: Supplementary file 1 [file biomedicines-14-00138-s001.zip › Supplementary file S2 coverage-genes fibrosi submitted.png]
